# Using auditory pre-information to solve the cocktail-party problem: electrophysiological evidence for age-specific differences

**DOI:** 10.3389/fnins.2014.00413

**Published:** 2014-12-10

**Authors:** Stephan Getzmann, Jörg Lewald, Michael Falkenstein

**Affiliations:** ^1^Aging Research Group, Leibniz Research Centre for Working Environment and Human Factors, Technical University of Dortmund (IfADo)Dortmund, Germany; ^2^Faculty of Psychology, Ruhr-University BochumBochum, Germany

**Keywords:** auditory perception, spoken language understanding, “cocktail-party” problem, aging, event-related potentials

## Abstract

Speech understanding in complex and dynamic listening environments requires (a) auditory scene analysis, namely auditory object formation and segregation, and (b) allocation of the attentional focus to the talker of interest. There is evidence that pre-information is actively used to facilitate these two aspects of the so-called “cocktail-party” problem. Here, a simulated multi-talker scenario was combined with electroencephalography to study scene analysis and allocation of attention in young and middle-aged adults. Sequences of short words (combinations of brief company names and stock-price values) from four talkers at different locations were simultaneously presented, and the detection of target names and the discrimination between critical target values were assessed. Immediately prior to speech sequences, auditory pre-information was provided via cues that either prepared auditory scene analysis or attentional focusing, or non-specific pre-information was given. While performance was generally better in younger than older participants, both age groups benefited from auditory pre-information. The analysis of the cue-related event-related potentials revealed age-specific differences in the use of pre-cues: Younger adults showed a pronounced N2 component, suggesting early inhibition of concurrent speech stimuli; older adults exhibited a stronger late P3 component, suggesting increased resource allocation to process the pre-information. In sum, the results argue for an age-specific utilization of auditory pre-information to improve listening in complex dynamic auditory environments.

## Introduction

Verbal communication under so-called “cocktail-party” conditions is one of the most amazing abilities of the human auditory system. When we are confronted with more than one acoustic stimulus at once (be it speech or non-linguistic stimuli), it is necessary (a) to perceptually segregate relevant auditory information from concurrent background sound and (b) to focus auditory attention on the source of interest (Cherry, 1953; Bronkhorst, 2000; for review, see Darwin, 2008). The aspect of segregation depends on the principles of auditory scene analysis (Bregman, 1990), in which a stream of auditory information is segregated and grouped into a number of perceptually distinct and coherent auditory objects. The aspect of attentional allocation is based on the ability of selectively focussing on an auditory object of interest. Studies on spoken language processing in multi-talker situations suggested that auditory object formation, object selection, and attentional allocation are closely related to each other, and can be described within a multiple-stage model of successful “cocktail-party” listening (Ihlefeld and Shinn-Cunningham, 2008; Shinn-Cunningham and Best, 2008).

There is evidence that listeners use different strategies to solve the “cocktail-party” problem. These strategies might become important especially in dynamic auditory environments, in which changing auditory objects require more effort in scene analysis and selective attention (Best et al., 2008). Either a more resource-allocating and effortful (“bottom-up”) strategy requiring increased allocation of processing resources and selective attention can be of advantage, or a more cognitive (“top-down”) strategy based on contextual and facilitatory information (Obleser and Kotz, 2011). In the latter case, prior knowledge comprising contextual information and expectations on the auditory scene is used to anticipate scene analysis and selective attention. For example, prior knowledge of the location of a target stimulus significantly improves listening performance compared with a condition in which spatial pre-information is absent (e.g., Kidd et al., 2005; Best et al., 2008; Singh et al., 2008; Kitterick et al., 2010; Kopčo et al., 2010). Also, voice characteristics (e.g., Brungart et al., 2001; Allen et al., 2008) and contextual pre-information is used to anticipate auditory scene analysis (e.g., Aydelott et al., 2010).

It is well-known that “cocktail-party” listening becomes more difficult in old age. On the one hand, these deficits result from age-related changes in cochlear, retrocochlear, and central auditory processing, i.e., declines in binaural and spectro-temporal analyses (for review, see Humes and Dubno, 2010; Fitzgibbons and Gordon-Salant, 2010; Humes et al., 2012). On the other hand, there is growing evidence that age-related declines in general cognitive abilities (i.e., working memory capacity, inhibitory control, and information processing speed; Van der Linden et al., 1999) affect the older listener's ability to understand language in the presence of competing speech (for review, see Burke and Shafto, 2008). In particular, it has been assumed that strategies used to compensate age-related declines in the auditory system may fail if cognitive resources decline (e.g., Pichora-Fuller, 2008; for review, see Schneider et al., 2010). The use of compensation strategies may come at a cost of successful perception (Wingfield and Tun, 2007), and deficits in “cocktail-party” listening may also result—at least in part—from reduced top-down processing capacities. As a consequence, contextual and facilitatory pre-information might be used less efficiently for completion of missing information in scene analysis and selective attention in older, than younger, adults.

In the present study, the use of prior knowledge was studied in a simulated dynamic “cocktail-party” situation. Younger and older adults performed a modified version of the “stock-price monitoring” task (Getzmann and Falkenstein, 2011): Sequences of company names and values were simultaneously presented by four different talkers who continuously changed their locations, and the subjects judged whether the value of a target company was above or below a given level. Thus, according to the above described multiple-stage model, the subjects firstly had to extract the relevant information from the dynamic auditory scene, and to determine whether the target company was actually present or not. Then, to adequately use the relevant target information, they had to focus auditory attention on the talker providing the target information, and to decide whether the value of the company was above or below the threshold. To differentiate between these processes two different performance measures were analyzed: (1) The *detection rate* indicated whether or not the subject was able to extract the target information (i.e., the company name) from the auditory scene; (2) the *discrimination rate* indicated whether or not the subject was able to subsequently focus on the talker of the relevant information, and to identify the company value. While detection required the mere recognition of the name of the target company (without determination of the talker and his or her location), discrimination was a substantially more complex process involving both the recognition of the identity of the target talker and the extraction of the relevant information from concurrent auditory input.

Three different types of pre-cues were presented: (1) a *linguistic cue* in which all the company names of the following stimulus in the sequence were pre-presented, (2) a non-linguistic *spatial cue* that indicated the position of the target company, and (3), as a baseline condition, a non-specific cue that only cued the onset of the speech stimuli. We consider the spatial cue as the most informative (allowing the subject to focus on the location of the target stimulus before it appeared), and the non-specific cue as the less informative (because it neither indicated the subsequent company name, nor the target location). The linguistic cue may have partly been informative as it enabled the subject to anticipate the auditory scene, i.e., to analyze whether or not the target stimulus was present in the subsequent trial, and to identify talker and location of the target. To clarify whether adults at different ages made equal use of these different cues in “cocktail-party” listening, detection and discrimination errors in the linguistic and spatial cue conditions were compared with those in the non-specific baseline condition. Furthermore, to assess possible differences in the underlying cortical processes, event-related potentials (ERPs) were analyzed. Effects of age and cue type on early stimulus processing should be indicated by the P1 and N1 deflections, while correlates of subsequent processing stages are given by the P2, N2, and P3 deflections. These later ERP components depend more on the listener's attentional state and reflect controlled processing on a higher level of perceptual and cognitive operations (e.g., Gaillard, 1988): While the fronto-central P2 has been related to attentional allocation (Potts, 2004), and the parietal P3 to the allocation of processing resources (Polich, 1986, 2007), the fronto-central N2 is assumed to be a correlate of cognitive control and inhibition of irrelevant information (Folstein and Van Petten, 2008). Thus, the neural correlates of successful speech-in-noise perception should become manifest by contrasting the ERPs for the different cue conditions. The comparison of the two age groups should allow conclusions on whether these neural processes vary as a function of age.

## Material and methods

### Subjects

Twenty-four young (12 female, mean age 26.4 years, age range 21–35 years) and 24 middle-aged and older (12 female, mean age 64.6 years, age range 57–74 years) adults took part in the study. The young participants were recruited from local colleges, the older participants through newspaper advertisements and flyers distributed in the city of Dortmund (Germany). All participants reported to be right-handed, without any known acute or chronic medical illness, free of medication, and without any history of neurological, psychiatric, or chronic somatic problems. To exclude confounding effects of profound clinically-relevant hearing deficits, all participants underwent standard pure-tone audiometry (Oscilla USB 330; Inmedico, Lystrup, Denmark) at 125–8000 Hz. Except mild to moderate presbyacusis in the older group, the audiograms of all subjects were within a defined tolerance zone, indicating normal hearing below 4000 Hz (thresholds better than 30 dB hearing loss). The subjects gave their written informed consent and were paid for participation. The study conformed to the Code of Ethics of the World Medical Association (Declaration of Helsinki) and was approved by the local Ethical Committee of the Leibniz Research Centre for Working Environment and Human Factors, Dortmund, Germany.

### Apparatus, stimuli and task

The experiment took place in a dimly lit, electrically shielded, and sound-proof room (5.0 × 3.3 × 2.4 m^3^) with pyramid-shaped foam acoustic panel on ceiling and walls, and a sound-absorbing woolen carpet on the floor. The ambient background noise level was below 20 dB(A) SPL. During the experiment, the subject was seated on a comfortable, vertically adjustable chair. The position of the head was held constant by a chin rest.

**Figure 1:**
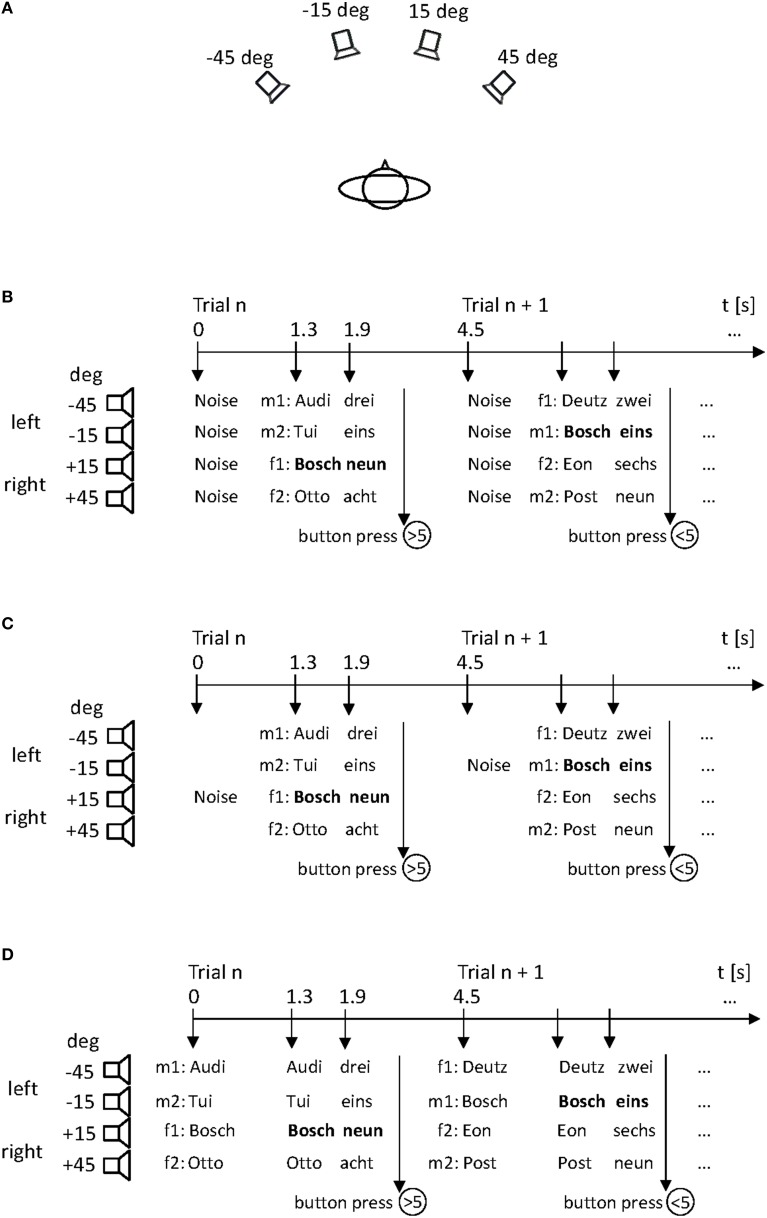
**The simulated “stock-price monitoring” scenario with four talkers (two male: m1, m2; two female: f1, f2) presenting sequences of short company names and numbers from different locations in space**. All stimuli were presented by four loudspeakers arranged in the subject's horizontal plane **(A)**. Sequences were either cued by noise bursts emitted from all four loudspeakers (**B**: non-specific cue), by a single noise burst indicating the spatial position of the target stimulus (**C**: spatial cue), or by speech stimuli corresponding to the company names (**D**: linguistic cue). The spatial positions of the talker s (m1, m2, f1, f2) changed from trial to trial. The subject responded to the value of a target company (here “Bosch”) that was either below (<5) or above (>5) a critical value.

The speech stimuli consisted of 8 one- to two-syllable components or abbreviations of German company names (Audi, Bosch, Deutz, Eon, Gerri, Otto, Post, Tui) and 8 one- to two-syllable German numerals (Eins [1], Zwei [2], Drei [3], Vier [4], Sechs [6], Sieben [7], Acht [8], Neun [9]). Stimuli were spoken by monolingual native German adults (two male, two female) of young and middle age without any dialect or speech disorders, and were digitally recorded in a sound-proof and anechoic environment using a freestanding microphone (MCE 91, Beyerdynamic, Heilbronn, Germany) and a mixing console (1202-VLZ PRO, Mackie, Woodinville, WA, sampling rate 48 kHz). The fundamental frequencies of the voices were 123 Hz and 126 Hz for the male talkers, and 162 Hz and 171 Hz for the female talkers. The sound stimuli were processed offline using CoolEdit 2000 (Syntrillium Software Co., Phoenix, AZ, USA). In addition to the speech stimuli, four white-noise bursts with rise/decay times of 50 ms were generated digitally. The duration of each type of sound stimulus was 500 ms. The sound level was about 65 dB(A). The sounds were converted to analog form via a computer-controlled external soundcard (Sound Blaster Audigy 2 NX, Creative Labs, Singapore). The stimuli were presented by four broad-band loudspeakers (SC 5.9, Visaton, Haan, Germany) that were mounted in front of the subject at a distance of 1.5 m from the center of the head (Figure 1A). The loudspeakers were arranged at ear level in the horizontal plane at −45°, −15° (left), +15°, and +45° (right). The loudspeakers were selected on the basis of similar efficacy and frequency response curves to minimize output and fidelity differences. They were controlled by custom-made amplifiers and software.

A speech perception task was employed, in which sequences of a company name and a numeral simulating its stock price (e.g., “Bosch—zwei” [“Bosch—two”] or “Deutz—acht” [“Deutz—eight”]) were recited. Each company name was randomly combined with one of the eight numerals. The combinations were presented following a pseudo-random scheme in which the locations of the talkers and the companies changed between trials. The task was to monitor the price of a target company (either “Bosch” or “Deutz,” balanced across subjects) while ignoring all other companies. The subjects were informed that the target company was present in 50% of trials, and that the target value was either below or above the value of five. They had to press the lower button of a response box when the price of the target company was below this value and the upper button when the price exceeded this value, using the index and the middle finger of the dominant hand. When subjects did not identify the target company, they were instructed to press no button; when subjects identified the target company, but not the target value, they were instructed to make a guess, i.e., to press either button.

A total of 1024 trials were presented in four blocks, each lasting about 20 min. The target company was present in 128 trials per block. Among these target trials, the target value was either smaller than five (values “1,” “2,” “3,” “4”; each in 12.5% of target trials) or larger than five (values “6,” “7,” “8,” “9”; each in 12.5% of target trials). The session began with a practice block, in which only one talker was active, i.e., a single sequence of company names and values was presented. The sequence was cued by 500-ms incoherent white-noise bursts emitted simultaneously from all four loudspeakers. Eight hundred milliseconds after the offset of this cue, the company name was presented that was followed by the numeral after a 100-ms silent interval. Each trial lasted 4.5 s, leaving 2.1 s after the end of the numerals for response. The practice block served to familiarize the participants with the task and as a control condition to test whether the subject was generally able to perform the task.

After the practice block, subjects completed three experimental blocks, in which four sequences of company names and values were presented simultaneously in each trial. Different company names and values were presented within one trial, with each talker located at a different position. In the baseline block, the sequences of company names and values were cued by noise bursts emitted from all four loudspeakers (non-specific cue; Figure 1B). In the spatial-cue block, the cue consisted of a single noise burst emitted from the loudspeaker where the target stimulus would later occur (*spatial cue;* Figure 1C). In the linguistic-cue block, the cue consisted of speech stimuli that corresponded to the company names of the subsequent stimulus pairs (company—number) (*linguistic cue;* Figure 1D). The three blocks were separated by a short rest break and counterbalanced across subjects. No feedback was given at any time during the experiment.

### Data recording and analysis

#### Behavioral data

Two different error measures were analyzed, the first one reflecting the failure to detect the target company among the concurring companies (the *detection* error) and the second one reflecting the failure to identify the value of the target company (the *discrimination* error). The detection error was defined as the percentage of trials in which a subject either missed the target company (in target trials) or produced false alarms (in non-target trials). An initial analysis of detection errors showed that these were mainly missing responses (20.5% on average), while false alarms were rare (1.2% on average). The discrimination error was defined as the percentage of target trials in which a subject correctly identified the target company, but not the company value (i.e., they pressed the false response button). Detection and discrimination errors were computed for each subject and condition, and subjected to Three-Way ANOVAs with between-subjects factors Age (younger, older) and within-subjects factors Cue (non-specific, linguistic, spatial) and Error type (detection, discrimination). To assess significant differences, a Bonferroni correction was applied; only the corrected probability values are reported. Levene's test was used to assess the homogeneity of variance, and the degrees of freedom were adjusted if variances were unequal. Effect sizes were computed to provide a more accurate interpretation of the practical significance of the findings, using the partial eta-squared coefficient (η^2^_*p*_).

#### EEG data

The continuous EEG was sampled at 2048 Hz using 64 electrodes and a BioSemi amplifier (Active Two; Biosemi, Amsterdam, Netherlands). Electrode positions were based on the International 10–10 system. The amplifier bandpass was 0.01–140 Hz. Horizontal and vertical eye positions were recorded by electro-oculography (EOG) using six electrodes positioned around both eyes. Two additional electrodes were placed on the left and right mastoids. Electrode impedance was kept below 10 kΩ. The raw data were downscaled offline to a sampling rate of 1000 Hz, digitally band-pass filtered (cut-off frequencies 0.5 and 25 Hz; slopes 48 dB/octave), and re-referenced to the linked mastoid electrodes. The data were segmented into 4400-ms stimulus-locked epochs covering the period from −200 to 4200 ms relative to cue onset. Data were then corrected for ocular artifacts using the Gratton and Coles procedure (Gratton et al., 1983). Individual epochs exceeding a maximum-minimum difference of 200 μV and a maximum voltage step of 50 μV per sampling point were excluded from further analysis (automatic artifact rejection as implemented in the BrainVision Analyzer software, Version 1.05; Brain Products, Gilching, Germany). The remaining epochs were baseline corrected to a 200-ms prestimulus window relative to the cue onset. Target trials containing correct responses were averaged for each listener; for the non-target trials, each epoch was averaged. Peaks of the different ERP deflections were defined as maximum positivity or negativity within particular latency windows of the specific waveforms (P1: 20–120 ms at FCz, N1: 60–160 ms at Cz, P2: 160–260 ms at FCz, N2: 250–345 ms at FCz, N400: 346–500 ms at FCz, P3: 300–800 ms at Pz, after cue onset). ERP peak latencies were measured at electrode positions chosen to be commensurate with previous knowledge of the topographical scalp distribution of specific ERPs (for review, Smith et al., 1980; Polich, 1986, 2007; Barrett et al., 1987; Näätänen and Picton, 1987; Lovrich et al., 1988; Friedman et al., 1993). The ERP latencies were subjected to ANOVAs with between-subjects factor Age and within-subjects factor Cue. Amplitudes were analyzed within an array of 3 × 3 electrodes around the vertex position (F3, Fz, F4, C3, Cz, C4, P3, Pz, P4). This resulted in two additional within-subjects factors: Frontality and Laterality. Amplitudes were subjected to Four-Way ANOVAs (Age, Cue, Frontality, and Laterality). For comparison of the cue conditions, Bonferroni-corrected *post-hoc t*-tests were applied.

## Results

### Behavioral data

**Figure 2:**
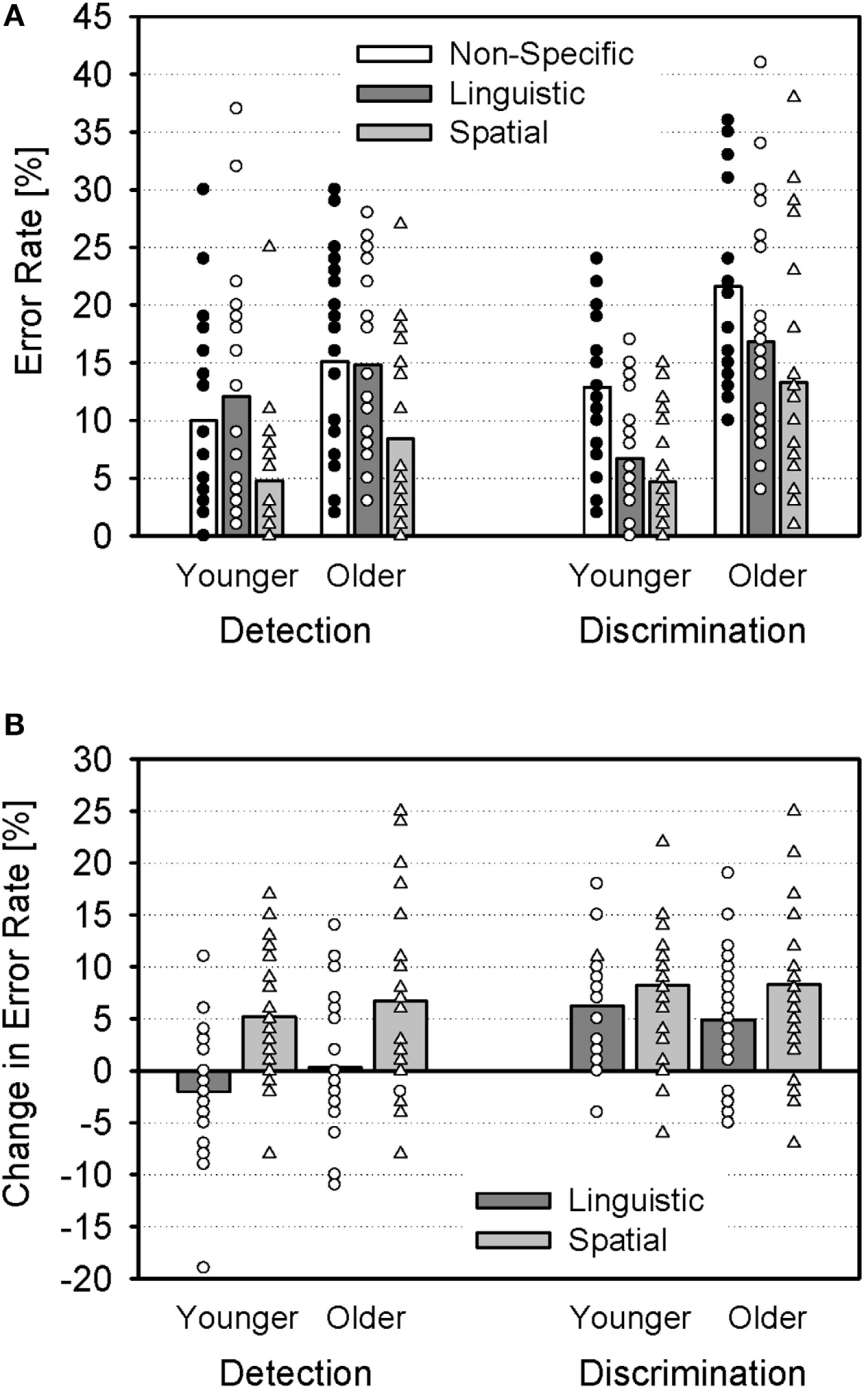
**Error rates (group means and individual values)**. **(A)** Detection errors (mean rate of trials in which subjects failed to detect the target stimulus, i.e., the company name) and discrimination errors (mean rate of target trials in which subjects failed to correctly identify the target value) for non-specific, linguistic, and spatial cues, shown separately for younger and older subjects. **(B)** Change in error rates for linguistic and spatial cues (relative to non-specific cues).

Detection and discrimination errors of each cue condition are shown in Figure 2A for the younger and older groups. There was a main effect of Age [*F*_(1, 46)_ = 19.67; *p* < 0.001; η^2^_*p*_ = 0.30], indicating more errors of older than younger subjects (15.0% vs. 8.5%). Also, there was a main effect of Cue [*F*_(2, 92)_ = 53.16; *p* < 0.001; η^2^_*p*_ = 0.54] and an interaction of Cue and Error [*F*_(2, 92)_ = 10.40; *p* < 0.001; η^2^_*p*_ = 0.18], indicating that the cues had differential effects on detection and discrimination errors. There were no interactions of Cue and Age [*F*_(2, 92)_ = 0.15; *p* > 0.05; η^2^_*p*_ < 0.01], Age and Error [*F*_(1, 46)_ = 3.64; *p* = 0.06; η^2^_*p*_ = 0.07], or Cue, Age, and Error [*F*_(2, 92)_ = 0.87; *p* > 0.05; η^2^_*p*_ = 0.02].

In order to further analyze the interaction of Cue and Error, additional Two-Way ANOVAs (between-subjects factor Age and within-subjects factor Cue) were conducted separately for both error types. For detection errors, the ANOVA indicated a significant main effect of Cue [*F*_(2, 92)_ = 27.29; *p* < 0.01; η^2^_*p*_ = 0.37]. *Post-hoc t*-tests (Bonferroni-corrected) revealed significant differences between non-specific and spatial cues and between linguistic and spatial cues (both *p* < 0.01), but not between non-specific and linguistic cues (*p* > 0.05; Figure 2). There was neither an effect of Age [*F*_(1, 46)_ = 3.69; *p* > 0.05; η^2^_*p*_ = 0.07], nor an interaction of Age and Cue [*F*_(2, 92)_ = 0.86; *p* > 0.05; η^2^_*p*_ = 0.02]. For discrimination errors, there was a significant main effect of Cue [*F*_(2, 92)_ = 36.12; *p* < 0.001; η^2^_*p*_ = 0.44], and *post-hoc t*-tests indicated that differences between cue conditions were significant in each case (*p* < 0.05). In addition, there was a significant main effect of Age [*F*_(1, 46)_ = 19.68; *p* < 0.001; η^2^_*p*_ = 0.30], but no interaction of Age and Cue [*F*_(2, 92)_ = 0.34; *p* > 0.05; η^2^_*p*_ < 0.01].

In sum, the behavioral data indicated (1) an overall better performance of younger, than older, subjects, (2) substantial improvements in performance of both age groups when specific cues were provided, and (3) a differential effect of linguistic and spatial cues on detection and discrimination errors: While the spatial cues improved both detection of the target stimulus and the discrimination of the target value, the linguistic cue improved discrimination (relative to the non-specific cues), but not detection (Figure 2B).

### ERP analysis of cue processing

**Figure 3:**
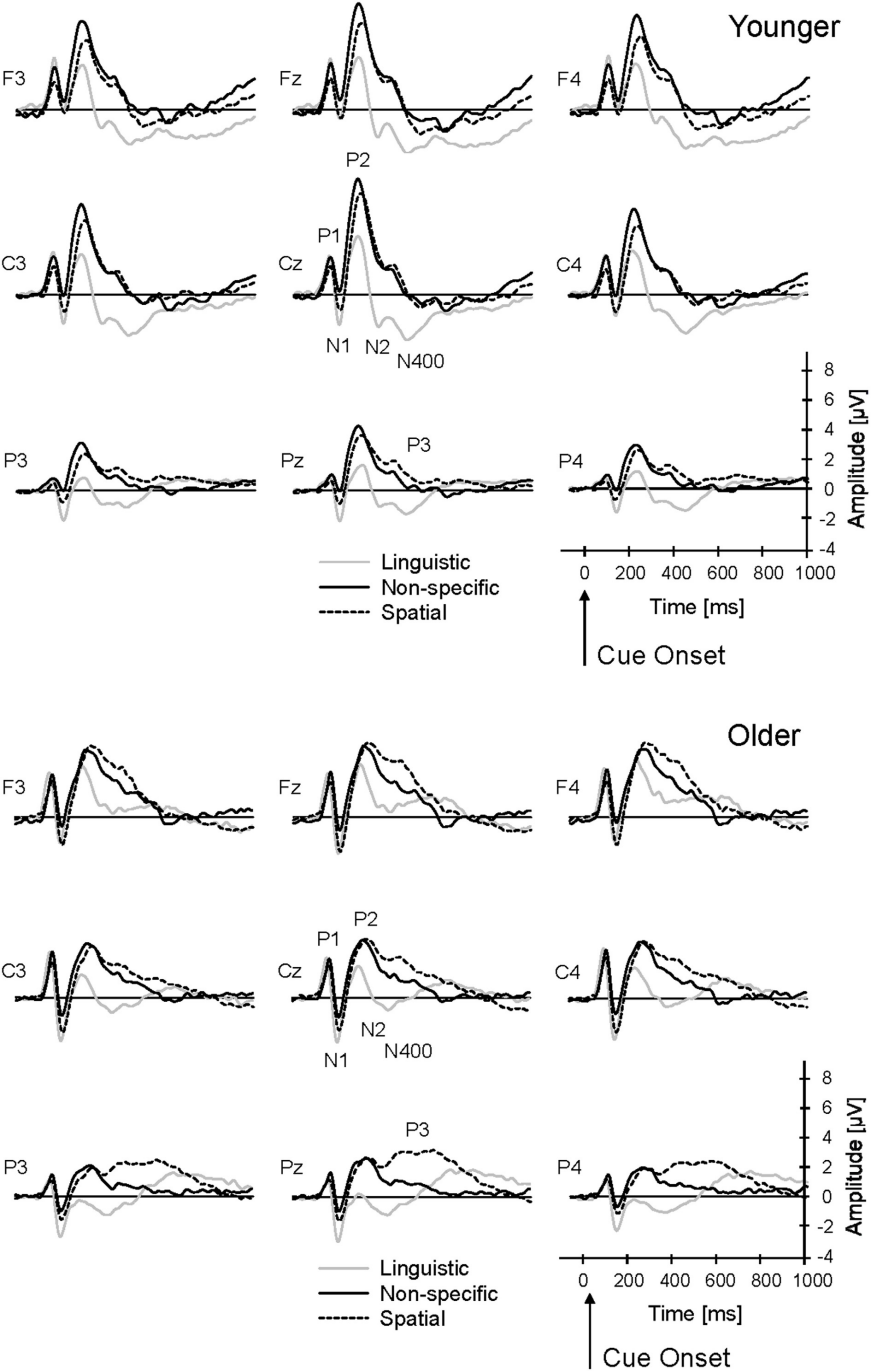
**Grand-average ERPs to cue stimuli (non-specific, linguistic, and spatial cues) for younger and older subjects**. The ERPs were averaged across target and non-target trials. P1, N1, P2, N2, N400, and P3 components were analyzed within a 3 × 3 array of frontal (F3, Fz, F4), central (C3, Cz, C4), and parietal (P3, Pz, P4) electrodes.

The cue onset produced a typical fronto-central P1-N1-P2 complex, peaking at about 69 ms, 112 ms, and 215 ms, respectively (averaged across groups, cue conditions, and target and non-target trials; Figure 3). In addition, the linguistic cue produced a pronounced fronto-central negativity that appeared to be double-peaked (N2: 312 ms; N400: 412 ms) in the younger group; this negative complex was reduced in the older group. In the older group, there was an additional late parietal positivity at about 638 ms (P3) after meaningful (spatial and linguistic) cues that appeared to be reduced in the younger group. In the following sections, these ERP components are compared, focussing on the effects of Age and Cue on ERP latencies, amplitudes, and topographies.

#### P1

The P1was larger with the non-specific (2.8 μV) and the linguistic cue (2.8 μV), relative to the spatial cue (2.2 μV) [main effect of Cue: *F*_(2, 92)_ = 13.2; *p* < 0.001; η^2^_*p*_ = 0.22; significant differences between non-specific and spatial cues, and between linguistic and spatial cues; *p* < 0.001]. Also, the P1 latency was shorter with the linguistic cue (62 ms) than with the spatial cue (69 ms) and the non-specific cue (75 ms) [main effect Cue: *F*_(2, 92)_ = 13.9; *p* < 0.001; η^2^_*p*_ = 0.23; significant differences between all cue conditions; *p* < 0.05]. There were no interaction of Age and Cue, and no main effect of Age on P1 amplitudes, while the older group had a slightly shorter P1 latency than the younger group [66 ms vs. 72 ms; *F*_(1, 46)_ = 4.2; *p* < 0.05; η^2^_*p*_ = 0.08].

#### N1

**Figure 4:**
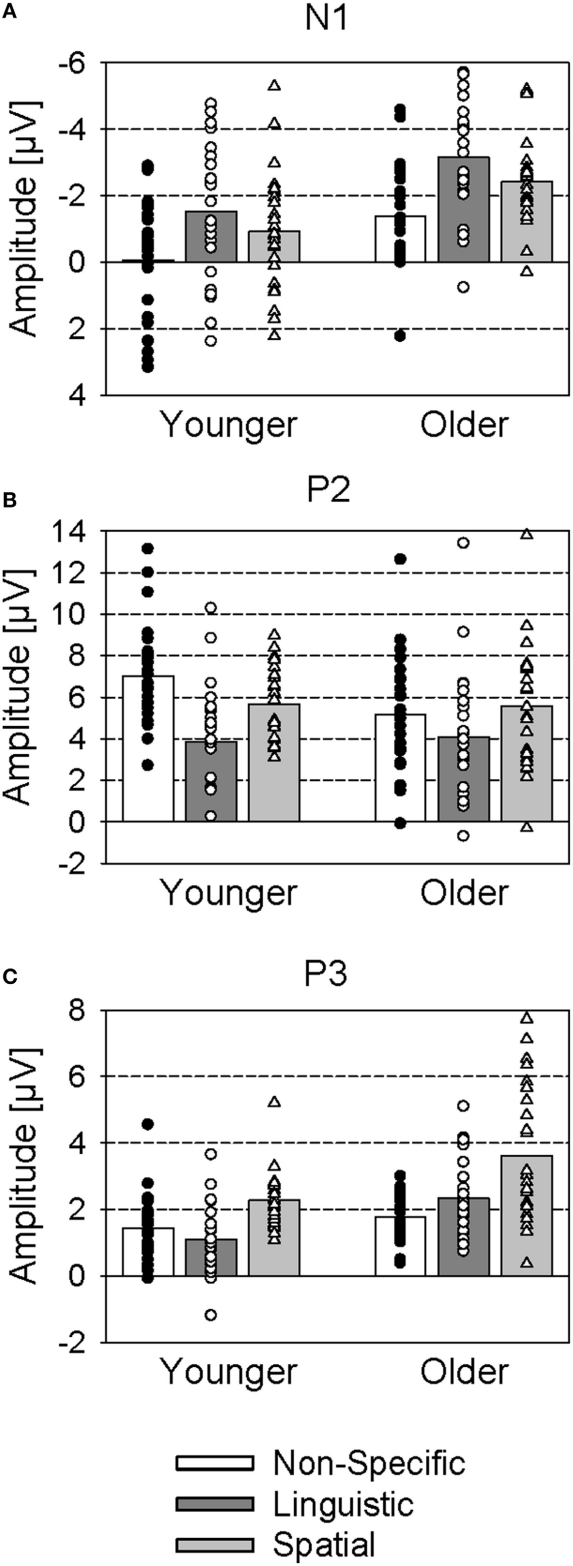
**Mean ERP amplitudes (group means and individual values) to cue stimuli (non-specific, linguistic, and spatial cues) for younger and older subjects**. **(A)** N1 (averaged across the 3 × 3 electrode array); **(B)** P2 (averaged across frontal electrodes F3, Fz, F4); **(C)** P3 (averaged across parietal electrodes P3, Pz, P4).

The N1 was most pronounced over the vertex position. It was largest with the linguistic cue (−2.3 μV), decreased with the spatial cue (−1.7 μV), and was nearly absent with the non-specific cue (−0.7 μV) [main effect Cue: *F*_(2, 92)_ = 39.2; *p* < 0.001; η^2^_*p*_ = 0.46; significant differences between all cue conditions; all *p* < 0.01; Figure 4A]. Also, older subjects had a larger N1 [−2.3 μV vs. −0.8 μV; *F*_(1, 46)_ = 10.9; *p* < 0.01; η^2^_*p*_ = 0.19] and later N1 [117 ms vs. 107 ms; *F*_(1, 46)_ = 7.3; *p* < 0.01; η^2^_*p*_ = 0.14] than younger subjects. No significant interaction of Age and Cue occurred.

#### P2

The P2 had a fronto-central topography and its amplitude was larger with non-specific cues (5.1 μV) and spatial cues (4.6 μV) than with linguistic cues (2.7 μV) [main effect Cue: *F*_(2, 92)_ = 51.6; *p* < 0.001; η^2^_*p*_ = 0.53; significant differences between linguistic and non-specific cues, and between linguistic and spatial cues; both *p* < 0.001]. These differences between cue conditions were most pronounced over the vertex electrode position, but decreased over lateral and parietal electrode positions [interaction of Cue and Laterality: *F*_(4, 184)_ = 10.9; *p* < 0.001; η^2^_*p*_ = 0.19; interaction Cue and Frontality: *F*_(4, 184)_ = 2.7; *p* < 0.05; η^2^_*p*_ = 0.06]. Also, younger subjects had a larger P2 than older subjects over the vertex position [7.0 μV vs. 4.6 μV; interaction of Age and Laterality: *F*_(2, 92)_ = 3.8; *p* < 0.05; η^2^_*p*_ = 0.08; interaction Age and Frontality: *F*_(2, 92)_ = 18.0; *p* < 0.001; η^2^_*p*_ = 0.28]. These age-related difference in P2 amplitude occurred mainly in the non-specific cue condition [interaction Age and Cue: *F*_(2, 92)_ = 3.3; *p* < 0.05; η^2^_*p*_ = 0.07] over the frontal electrode positions [interaction Age, Cue, and Frontality: *F*_(4, 184)_ = 5.9; *p* < 0.001; η^2^_*p*_ = 0.11]: While the younger group had a larger P2 than the older group in the non-specific condition, the groups did not differ from each other in the linguistic and spatial cue conditions (Figure 4B).

The P2 latency was larger with the spatial cue (229 ms) than with the non-specific cue (212 ms) and the linguistic cue (205 ms) [main effect Cue: *F*_(2, 92)_ = 25.5; *p* < 0.001; η^2^_*p*_ = 0.36; significant differences between spatial and non-specific cues, and between spatial and linguistic cues; both *p* < 0.001]. Also, the latency was increased in the older subjects (227 ms) relative to the younger subjects (203 ms) [main effect Age: *F*_(1, 46)_ = 35.2; *p* < 0.001; η^2^_*p*_ = 0.43].

#### N2

A clear N2 was only evident in the linguistic cue condition. A Three-Way ANOVA was therefore conducted with Age as between-subjects factor, and Frontality and Laterality as within-subjects factors. The N2 was most prominent over the fronto-central electrode positions. Younger subjects had a larger N2 than the older ones [−1.9 μV vs. −0.8 μV; main effect Age: *F*_(1, 46)_ = 5.3; *p* < 0.05; η^2^_*p*_ = 0.10], especially over frontal and central positions [interaction Age and Frontality: *F*_(2, 92)_ = 16.1; *p* < 0.001; η^2^_*p*_ = 0.25]. No differences occurred in N2 latency.

#### N400

The N400 was evident as a second negative peak following the N2 in the linguistic cue condition. As the N2, the N400 had a fronto-central topography and was most prominent in the younger subjects. Thus, a Three-Way ANOVA (Age, Frontality, and Laterality) indicated a stronger N400 in younger than older subjects [−2.8 μV vs. −0.8 μV; main effect Age: *F*_(1, 46)_ = 22.7; *p* < 0.001; η^2^_*p*_ = 0.33], and this difference was most pronounced over fronto-central areas [interaction Age and Laterality: F_(2, 92)_ = 8.2; interaction Age and Frontality: *F*_(2, 92)_ = 22.1; both *p* < 0.001; both η^2^_*p*_ = 0.15]. No differences occurred in N400 latency.

#### P3

There was a late positivity over parietal areas that was evident especially in the older group, and smaller in the younger group. A Three-Way ANOVA (Age, Cue, and Laterality) conducted over the parietal electrodes indicated larger amplitudes in older, than younger, subjects [2.6 μV vs. 1.6 μV; main effect Age: *F*_(1, 46)_ = 13.0; *p* < 0.001; η^2^_*p*_ = 0.22; Figure 4C], and larger amplitudes with the spatial cue (3.0 μV) than with the non-specific cues (1.6 μV) and the linguistic cues (1.7 μV) [main effect Cue: *F*_(2, 92)_ = 28.4; *p* < 0.001; η^2^_*p*_ = 0.38; significant difference between spatial and non-specific cues, and between spatial and linguistic cues; *p* < 0.001]. These differences were especially pronounced over the midline electrode position [interaction Cue and Laterality: *F*_(4, 184)_ = 4.50; *p* < 0.01; η^2^_*p*_ = 0.09]. Also, the amplitude differences between younger and older subjects were stronger with the spatial and linguistic cue than with the non-specific cue [interaction Age and Cue: *F*_(2, 92)_ = 4.05; *p* < 0.05; η^2^_*p*_ = 0.08]. Accordingly, additional Two-Way ANOVAs (Age and Laterality) indicated significantly higher P3 amplitudes of the older subjects relative to the younger one with the spatial cue [*F*_(1, 46)_ = 8.40; *p* < 0.01; η^2^_*p*_ = 0.15] and the linguistic cue [*F*_(1, 46)_ = 15.72; *p* < 0.001; η^2^_*p*_ = 0.26], but not the non-specific cue [*F*_(1, 46)_ = 1.76; *p* > 0.05; η^2^_*p*_ = 0.04].

The latency of P3 was smaller with the spatial cue (447 ms) and the non-specific cue (463 ms) than with the linguistic cue (658 ms) [main effect Cue: *F*_(2, 92)_ = 47.8; *p* < 0.001; η^2^_*p*_ = 0.51; significant difference between spatial and linguistic cues, and between the non-specific and linguistic cues; both *p* < 0.001]. Also, there was a trend to larger P3 latencies in older than younger subjects [548 ms vs. 497 ms; *F*_(1, 46)_ = 3.94; *p* = 0.053; η^2^_*p*_ = 0.08].

#### Comparison of target and non-target linguistic cues

**Figure 5:**
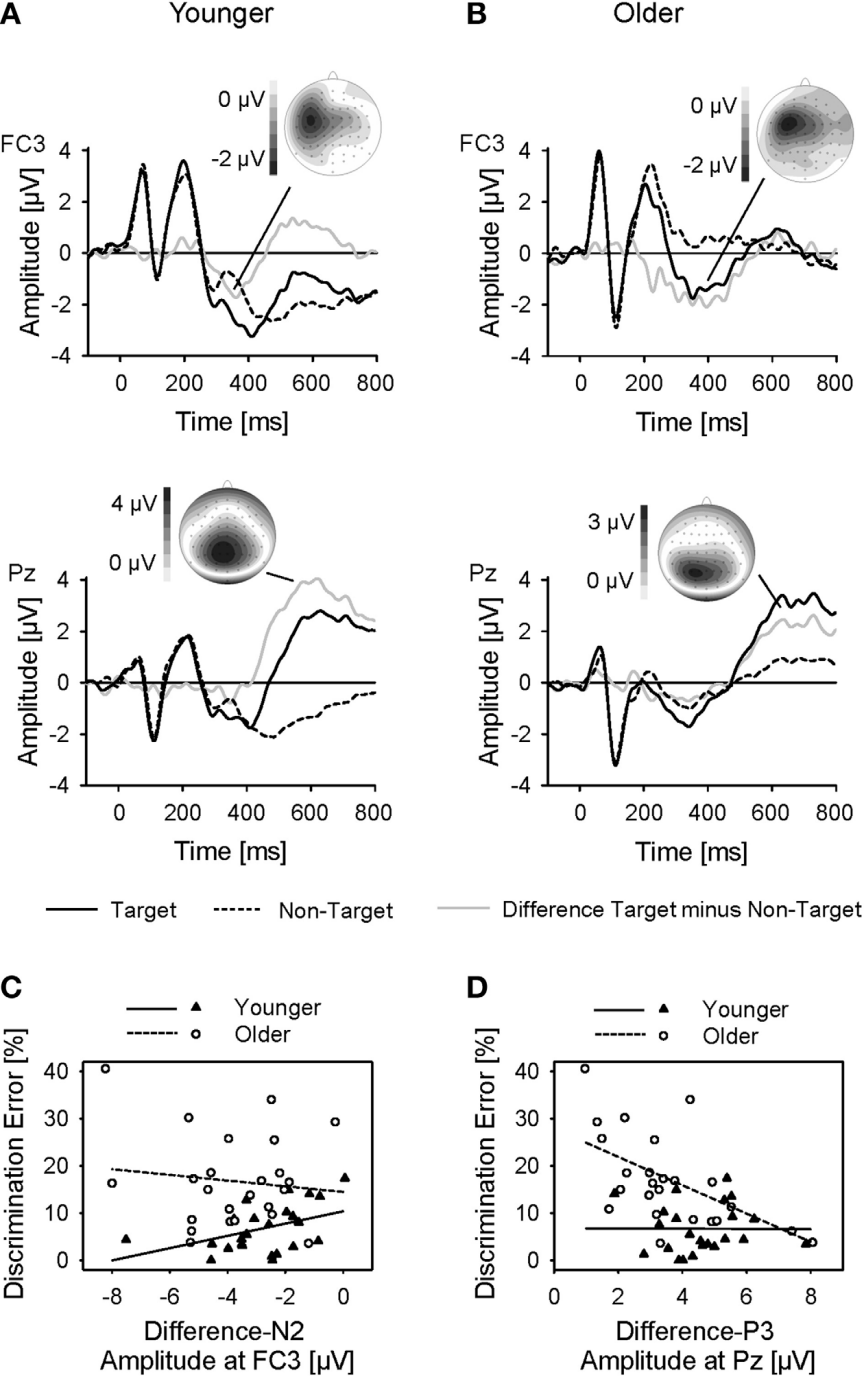
**(A,B)** Grand-average ERPs to the linguistic cue, plotted at electrode positions FC3 and Pz as a function of time relative to the cue onset for younger and older subjects, and for target-trials and non-target trials. In addition, difference waveforms and topographies of the difference-N2 and difference-P3 are plotted. **(C,D)** Difference-N2 amplitudes at FC3 and difference-P3 amplitudes at Pz of younger and older subjects plotted against rates of discrimination errors, with linear regression lines.

In contrast to the non-specific and the spatial cue conditions, the linguistic cue indicated whether the target stimulus was present in the following sequence (i.e., in target trials) or not (i.e., in non-target trials). To further test whether the two age groups differed in the usage of this information, the difference waveforms (target trials minus non-target trials) were computed, and difference ERPs to linguistic cues in target minus non-target trials were analyzed using Three-Way ANOVAs (Age, Frontality, Laterality). While differences in the P1-N1-P2 complex were rather marginal, the N2 appeared to be larger in target, than non-target, trials (Figure 5A). This difference-N2 was most pronounced over left-hemispheric fronto-central areas [main effect Frontality: *F*_(2, 92)_ = 23.62; main effect Laterality: *F*_(2, 92)_ = 19.39; both *p* < 0.001; both η^2^_*p*_ > 0.30]. There was no difference between age groups, neither in amplitudes nor latencies (both *p* > 0.05).

In order to assess whether the negativity found in the difference ERPs was related with performance, correlations between the amplitude of the difference-N2 (measured at its maximum, FC3) and the detection and discrimination errors were computed, separately for the younger and older subjects. There was a significant correlation between the difference-N2 amplitude and the rate of discrimination errors in the younger subjects (*r* = 0.43; *p* < 0.05; Figure 5C), but not in the older subjects (*r* = −0.12; *p* > 0.05). The correlation of the younger subjects was significantly higher than that of the older subjects (Fisher's *Z* = 1.87, one-tailed *p* < 0.05). Thus, superior performance in discrimination of the younger subjects came along with stronger left-frontal activation in target trials, while performance of the older subjects was not related to left-frontal activation. No significant correlations occurred between difference-N2 amplitude and detection errors (younger: *r* = −0.15; older: *r* = 0.20; both *p* > 0.05).

The analysis of the difference waveforms also revealed a pronounced difference-P3 that was maximal over midline-parietal areas [main effect Frontality: *F*_(2, 92)_ = 53.20; main effect Laterality: *F*_(2,92)_ = 10.82; both *p* < 0.001; both η^2^_*p*_ > 0.19; Figure 5B]. There was no effect of age on difference-P3 amplitudes, but latencies were larger in the older, than younger, group [661 ms vs. 610 ms; main effect Age: *F*_(1, 46)_ = 4.32; *p* < 0.05; η^2^_*p*_ = 0.09]. A correlational analysis indicated a significant correlation between the amplitude of difference-P3 (measured at Pz) and the rate of discrimination errors in the older subjects (*r* = −0.53; *p* < 0.01; Figure 5D), but not in the younger subjects (*r* = −0.01; *p* > 0.05). The correlation coefficient of the older subjects was significantly higher than that of the younger ones (Fisher's *Z* = 1.90, one-tailed *p* < 0.05). Higher performance in discrimination was thus correlated with stronger parietal activation in target trials in older subjects, but not in younger subjects. No significant correlations occurred between difference-P3 amplitude and detection errors (younger: *r* = −0.29; older: *r* = −0.10; both *p* > 0.05).

#### Correlational analysis of audiometric thresholds and ERP data

Although all listeners had clinically normal peripheral hearing, the hearing thresholds were significantly higher in the older group: There was a threshold difference between the age groups [younger: 9.4 dB nHL; older: 24.2 dB nHL; *t*_(46)_ = 8.85; *p* < 0.001] in the range from 250 to 4000 Hz (averaged across both ears). Moreover, there was a significant correlation of age and hearing thresholds (*r* = 0.83; *p* < 0.001). In order to estimate the extent to which the differences in cue processing could be based on deficits in peripheral hearing, the relationship between individual hearing thresholds and ERP data was analyzed by calculating partial correlations (i.e., correlations between hearing thresholds and ERP data when the effect of age was removed) for each peak amplitude, averaged across cue conditions. None of these correlation coefficients reached statistical significance (P1 at FCz: *r* = −0.08; N1 at Cz: *r* = 0.16; P2 at FCz: *r* = 0.06; N2 at FCz: *r* = 0.07; N400 at FCz: *r* = 0.18; P3 at Pz: *r* = 0.25; all *p* > 0.05).

## Discussion

Older participants showed significantly more discrimination errors than younger adults, while there was no difference in detection error rate between age groups. This observation is in line with everyday experiences of elderly who often report that they can *hear* spoken words, but cannot *understand* what has been said. In the present stock-price monitoring task, the older adults had less problems with the mere detection of the target company name, but they performed significantly worse than the younger group in the more complex discrimination task, in which they firstly had to determine the talker of the target stimulus, then to focus their attention on this talker, and finally to identify the target company value among the concurrent values. In contrast to the preceding study, in which significant differences in performance between younger and middle-aged adults were not found (Getzmann and Falkenstein, 2011), the present experimental settings thus seemed to be better suited to unveil age-related deficits in speech understanding. Here, permanent and rapid changes in the multiple-talker setting may have put much higher demands on speech processing than the rather steady listening situation in the previously. Spatial continuity has been found not only to avoid costs associated with switching the focus of spatial attention, but also to produce refinements in the spatial selectivity of attention across time (Best et al., 2008). Assuming higher costs of switching in older adults, it appears plausible that age-related differences are more pronounced in a dynamic listening situation, as was simulated here.

Regardless of these differences in performance, both age groups benefited from auditory pre-information. This is in line with previous studies (MacDonald et al., 2008; Sheldon et al., 2008; Ezzatian et al., 2011; for review, see Pichora-Fuller, 2008). The spatial cue improved both detection of the target stimulus and discrimination between company values, relative to the non-specific cues. As the spatial cue reliably indicated the location of the subsequent target stimulus, it enabled the participants to focus their auditory attention on the position of the target stimulus before it was present. The benefit given by this spatial pre-information is in accordance with previous studies on auditory spatial attention that demonstrated higher performance when pre-cueing the task-relevant location (Mondor and Zatorre, 1995; Kidd et al., 2005; Best et al., 2008; Singh et al., 2008). The linguistic cue also improved discrimination. When the company names of the following sequence was pre-presented, the participants received information on whether the target company would be present in the subsequent trial, and on the talker and location of that target stimulus. Thus, within the theoretical framework of a multiple-stage model of successful “cocktail-party” listening (Ihlefeld and Shinn-Cunningham, 2008; Shinn-Cunningham and Best, 2008), the linguistic cue may have triggered both auditory scene analysis and attention focussing on the target name, and thus improved discrimination between company values (relative to the non-specific cue). Its effectiveness did, however, not reach that of the spatial cue that allowed for the direct focussing of attention on the target location. In this context, it is important to note that the linguistic cue (as a cue to target location) was probably not as effective as the spatial cue, given that four stimuli were presented simultaneously in the former condition, while a single noise stimulus was presented in the latter condition (cf., e.g., Zündorf et al., 2011; Lewald and Hausmann, 2013). These differences in stimulus features may have contributed to the reduced effect of the linguistic cue, relative to the spatial cue. Interestingly, the linguistic cue did not improve the genuine detection of the target stimulus. A possible reason could be that the participants incompletely analyzed the sequence of company names following the linguistic cue: Given that the cue conditions were presented in a blockwise manner, the participants could have used the linguistic cue to determine whether or not the target company would be present in the ongoing trial, while leaving this information rather unexploited in the subsequent stimulus sequence. Thus, while primarily focussing on company values, they might have benefitted less from the double presentation of the company names.

The ERPs showed effects of cue condition and age. The early stimulus-related component P1 was smaller for the spatial than for the other cues, which can be explained by the lower loudness of the spatial cues, being single stimuli, compared to the multiple-stimuli nature of the other cue types. The precise perceptual processes reflected by the auditory P1 are still not fully understood. The P1 is elicited very early in the auditory perceptual processing stream (e.g., Grunwald et al., 2003), and is mainly driven by physical characteristics of the auditory stimulus. As such, the P1 amplitude has been shown to increase with increasing stimulus loudness, at least at low and moderate sound levels (Schechter and Buchsbaum, 1973). There was no effect of age on the P1, suggesting that this relatively early stage of auditory analysis is largely independent of age.

The N1 was largest for linguistic cues, smaller for spatial cues, and smallest for non-specific cues. These differences might be attributed—at least in part—to the physical differences between the stimuli, given that the spatial and non-specific cues consisted of broad-band noise while the linguistic cues consisted of speech stimuli. However, the N1 differences could also be interpreted in a way that the early auditory processes are enhanced by information bearing cues, in particular the most complex information, as in linguistic cues. Accordingly, it is known that the N1 is not only a correlate of early sensory processing, but also indicates allocation of attentional resources (Hillyard et al., 1973; for reviews, Luck et al., 2000; Eimer, 2014). Within the theoretical framework of an early selection model of attention, a larger N1 amplitude to relevant perceptual information may reflect a sensory gating mechanism of attention, facilitating subsequent processing of the stimulus. Importantly, the N1 was larger in older participants, irrespective of cue condition. This suggests that older adults invest more attentional resources in the processing of potentially relevant stimuli, such as the cues in the present study. In line with earlier findings (e.g., Yordanova et al., 2004), this could reflect compensatory strategies used by the older subjects. Taken together, it appears as if the second processing stage detectable in the ERP (N1) can be modulated by older subjects, while the preceding one (P1) cannot.

The P2 was reduced for the linguistic cues, relative to the other cues. While the younger group had a larger P2 than the older group in the non-specific condition, there was no group difference in the linguistic and spatial cue conditions. Assuming the P2 to be a correlate of attentional allocation (Potts, 2004), these data are hard to explain when seen in isolation. It does not seem plausible that more complex cues, such as the linguistic cues used here, require less allocation of attention than non-specific cues. It is more likely that the reduction of the P2 after the information-bearing linguistic cue is due to the overlap of the subsequent negative ERP complex (see below).

This negative complex, consisting of an early N2 and later N400, was indeed only seen after linguistic cues and it was larger in younger, than older, subjects. The N2 is known to reflect control processes in general (for reviews, see Patel and Azzam, 2005; Folstein and Van Petten, 2008) and has been related to conflict processing or inhibitory control of irrelevant information (e.g., Falkenstein et al., 2002; Melara et al., 2002; Bertoli et al., 2005). In the present context, the deep processing of the compound stimulus and/or the inhibition of the concurrent speech stimuli is necessary after linguistic cues. On the other hand, sub-components of the N2 have been also related to attentional processes, in particular, attention allocation to relevant stimulus features and detection of novelty, or mismatch from a perceptual template (for review, see Folstein and Van Petten, 2008). Thus, the larger N2 for linguistic cues, relative to the other cues, might also be attributed to the detection of the target stimulus. In line with this interpretation, the N2 was larger in target, than non-target, trials (see below). The N400 is a correlate of processing of meaningful (or potentially meaningful) stimuli that is often linked to language processing. It has been related to a wide range of cognitive functions, comprising orthographic and phonological analysis such as word recognition, integration of a word's meaning into the preceding context, as well as activation of access to semantic memory within a comprehension network (for review, see Kutas and Federmeier, 2011). There is evidence that both N2 and N400 decrease in amplitude in elderly. The reduction of N2 amplitude observed here is in line with previous findings (e.g., Anderer et al., 1996; Wascher et al., 2011; Wascher and Getzmann, 2014; Getzmann et al., 2015). It might indicate a less efficient control over the concurrent speech information in the older group which could be related to a general inefficiency to suppress neural activity associated with irrelevant and distracting information (for review, see Gazzaley and D'Esposito, 2007), according to the inhibitory deficit hypothesis (Hasher and Zacks, 1988). Alternatively, the reduced N2 amplitude might indicate age-related deficits in the detection of the target stimulus among the concurrent speech stimuli. It should be noted, however, that there were no differences in detection errors between age groups. In particular, after the linguistic cue, the age groups differed in discrimination errors rather than in detection errors (cf. Figure 2A). The decrease of N400 amplitude has also been found in a semantic categorization task (Kutas and Iragui, 1998) and could indicate reduced speech processing and—in particular—word recognition in the older group.

The N2 was larger after target than non-target cues. Accordingly, the analysis of the difference-ERP revealed that both age groups showed a pronounced negativity that had its maximum over the left-hemispheric speech processing area. Most importantly, the target-specific enhancement of the difference-N2 was related to discrimination errors in the younger group, indicating that larger amplitudes came along with better performance. There was no correlation of difference-N2 and detection errors, suggesting that the difference-N2 is not a correlate of mere target detection, but is associated with processes of scene analysis. Importantly, a significant correlation did not occur in the older group, but only in younger subjects. In concert with the overall stronger N2 component, the association of difference-N2 and discrimination performance could be interpreted in a way that the younger subjects used left-hemispheric speech areas for analysis of the linguistic cue. This is in line with previous studies, in which the role of linguistic and semantic context for speech comprehension under adverse conditions was investigated in younger adults (Davis and Johnsrude, 2003; Hannemann et al., 2007; MacDonald et al., 2008; Obleser and Kotz, 2010). Brain areas involved in compensating for distortion have been found mainly in the left hemisphere, partially overlapping with fronto-temporal language networks. In particular, areas in which activation correlated with intelligibility of distorted speech stimuli were located in left superior and middle temporal gyri, left inferior frontal gyrus, and left hippocampus (Davis and Johnsrude, 2003; Hannemann et al., 2007; MacDonald et al., 2008; Obleser and Kotz, 2010).

In older subjects, the N2 complex in general was reduced. Rather, the older subjects exhibited a late parietal P3 that was seen after both linguistic and spatial cues. The parietal component of the P3 is usually related to the allocation of processing resources that occurs independently of task and modality (Polich, 1986, 2007). The enhanced parietal P3 therefore suggests that older subjects allocated increased and speech-unspecific processing resources to the pre-information. This resource allocation process was delayed for the more difficult linguistic cue. Moreover, the analysis of target and non-target linguistic cues revealed a pronounced difference-P3 over parietal brain areas, resulting from a stronger parietal activation in target, than non-target, trials. The difference-P3 occurred in both age groups, and was—like the difference-N2 of the younger group—related to discrimination, but not detection, performance in the older subjects. Thus, it appears as if younger and older subjects allocated processing resources when the target stimulus was present. However, only in the older group the target-specific enhancement of the P3 was related to discrimination performance: Older subjects with a stronger difference-P3 performed better than those with a weaker difference-P3. This pattern of results could be interpreted within the theoretical framework of a compensation approach (for review, see Schneider et al., 2010), in which deficits in speech perception resulting from peripheral and central auditory processing are compensated by increased allocation of general processing resources.

In this context, it has to be mentioned that aging usually results in a desynchronization of correlated activity of neurons generating the different ERP peaks, and that age-related declines in neural synchrony have been found to contribute to changes in ERP amplitudes (e.g., Harris et al., 2014). Thus, the reduction of N2 and N400 amplitudes in the older group might—at least in part—be related to reduced neural synchrony. On the other hand, assuming an overall effect of desynchronization, one might speculate that amplitudes in the older group were rather underestimated, relative to the younger group. Given that we did not found an overall reduction of ERP amplitudes in the older group, but even greater amplitudes of N1 and P3, it could be that the processes reflected by these components were even more effective in the older group. In particular, increased compensatory activity might even be higher than indicated by the present differences in ERP amplitudes. Future studies using elaborative analysis techniques (e.g., single-trial analyses) will be neccessary to address the issue of neural desynchronization and its effect on age-related changes in cognitive processing.

Taken together, younger and older subjects obviously used different mechanisms to extract and process information of the linguistic cue: both groups analyzed this cue (which was indicated by a pronounced difference-N2 and difference-P3), but while the younger subjects mainly relied on early processing and inhibition of the speech stimulus, as reflected in the N2, the older subjects used an unspecific mechanism of resource allocation, as reflected in the P3. In sum, the results argue for an age-specific use of auditory pre-information to improve listening in complex dynamic auditory environments. However, the late mechanism (reflected by the P3 component), preferentially recruited by the older adults, was less effective than the earlier mechanism (reflected by the N2 component) which was preferentially used by younger adults.

### Conflict of interest statement

The authors declare that the research was conducted in the absence of any commercial or financial relationships that could be construed as a potential conflict of interest.
